# Qualitative insights on emergency preparedness and response to marburg virus disease in Ghana: The role of risk communication and community engagement

**DOI:** 10.1371/journal.pone.0309889

**Published:** 2024-12-12

**Authors:** Herman Nuake Kofi Agboh, Grace Adjei-Okai, George Adjeisah Adjei

**Affiliations:** 1 CHERF: Child Health and Educational Research Foundation, Accra, Ghana; 2 Catholic Health Service Trust, Accra, Ghana; Universite de Parakou, BENIN

## Abstract

**Objectives:**

Faith-based healthcare providers have played pivotal roles in recent public health responses to disease outbreaks, such as Ebola, COVID-19, and Marburg Virus Disease. However, the literature on their performance remains scarce. This research therefore evaluates the risk communication and community engagement capacity of the Christian Health Association of Ghana (CHAG) during the Marburg Disease Virus outbreak in Ghana.

**Method:**

Data were obtained from 15 clinical and nonclinical health workers affiliated with CHAG and the Ghana Health Service (GHS). Online interviews were conducted to assess the coordination of risk communication and community engagement during Marburg Virus outbreak in Ghana. Thematic analysis was employed for data analysis.

**Findings:**

Active engagement of national-level stakeholders, including the Ministry of Health and the Ghana Health Service, was observed. Outreach activities encompassing surveillance and contact tracing were also executed. However, resource constraints led to passive involvement of frontline workers in stakeholder meetings and risk communication activities, posing a limitation to the Risk Communication and Community Engagement (RCCE) effort.

**Conclusion:**

To address health system vulnerabilities and misinformation in low-resourced countries during health emergencies, a bottom-up approach is vital. This approach will enhance the capacity of communities, professionals, NGOs, and media to counter infodemics and disinformation. Government and healthcare facility owners must ensure robust logistical and policy preparations to effectively equip healthcare facilities for future disease outbreaks.

## Introduction

Risk Communication and Community Engagement (RCCE) is a vital public health tool for robust preparedness and response to disease outbreaks in Africa [1, 2]. According to the World Health Organization (WHO) [2, 3], RCCE is an indispensable component of the preparedness and emergency response system and essential for breaking the chain of disease transmission. It is a mechanism for transmitting healthcare and wellness information to individuals and communities for protection against health risks, ensuring the effective management of outbreaks, and lessening the undesirable effects of outbreaks on human life, health system infrastructure and commerce [1]. Documented evidence on RCCE is common, with many of these connected to recent disease outbreaks in Africa [3–5].

In the COVID-19 Strategic Preparedness and Response Plan for the WHO African Region (1 February 2021–31 January 2022) [3, 5], WHO utilised RCCE to disseminate timely, credible and relevant information to manage infodemic, and reduce the adverse repercussions of COVID-19 pandemics on individuals and communities. Similarly, the UNICEF Eastern and Southern Africa Regional Office’s report on “Risk Communication and Community Engagement for Ebola Virus Disease Preparedness and Response”, described RCCE as a complex system comprising the media, social mobilization and feedback systems, multilevel coordination mechanisms, and evidence-based strategies to understand community needs, fears, and concerns [4, 6]. Given its complexity, UNICEF stressed comprehensive community engagement as a vital approach to influencing people’s behaviours and willingness to accept public health and preventive measures.

Community engagement is a bottom-up strategy directed at involving individuals, groups and communities in the decision-making, planning, design, governance, and execution of disease preparedness and response plans [7, 8]. In Sierra Leone where 33 Ebola cases were detected, for instance, community-led interventions, including surveillance, mobile health interventions (mhealth), survivor reintegration programmes, health education through video and non-video information sharing platforms, point-of-care diagnostics, community quarantine, and the media, among others, were adopted to educate communities on the risks and preventive measures of Ebola [9]. According to Gilmore, et al [9], the effective implementation of social action during the outbreak of Ebola in West Africa contributed significantly to popular buy-in from Sierra Leoneans, facilitated indigenous ownership of the preparedness and response interventions used, and ensured the sustainability of other public health response interventions implemented. Similarly, in 2020, the Angolan government used RCCE to counter the rise of COVID-19 infodemics by tracking and analysing conversations on social and traditional media, providing periodic updates about the pandemic on national websites, and using posters and infographics to educate those who could not access the electronic messages [10]. In like manner, RCCE strategies were employed to manage the COVID-19 pandemic in Ghana. Some of these include executive briefings by the President, the Minister of Information, the media, and community champions [10]. Coupled with home visits, surveillance and contact tracing, billboard advertisements, emergency lines, pocket cards and civic education systems, Ghana was adjudged one of the countries with strong public health and risk communication structures in Africa [10].

This notwithstanding, it was observed that evidence on RCCE in Africa showing effective utilization of limited resources to prevent rapid transmission of infectious diseases also revealed concerning gaps in the emergency preparedness and response capacities of faith-based healthcare organizations (FBHOs) on the continent [11]. According to Boulenger, et al. [11], faith-based health delivery organizations in Africa are constrained with structural and nonstructural limitations, hindering their ability to respond to disease outbreaks [12]. Weak institutional frameworks, scarce resources, and limited disease surveillance structures, including the lack of qualified human resources, budgetary constraints and limited laboratory and testing capacities render FBHOs vulnerable to disease resurgence [13, 14]. Following a series of outbreaks between 2002 and 2020, comprising Ebola, H1N1, and COVID-19, on July 7, 2022, Ghana declared the outbreak of Marburg Virus Disease (MVD) in one of its faith-based institutions [12, 15]. Available reports on the management of the disease commended the country for preventing the rapid spread of MVD beyond the initial cases recorded but also exposed the inadequacy of the FBHO to handle the outbreak without substantial assistance from external partners [16, 17]. Additionally, literature on RCCE in FBHOs in Ghana is rare to come by [15], limiting the comprehensive understanding of the extent of their public health needs. This knowledge gap limits the capacity of policymakers and development partners to plan, organize and spearhead the effective execution of RCCE programmes to strengthen the preparedness and response capacities of FBHOs in Ghana [11, 15].

Given that FBHOs constitute about 40% of Ghana’s healthcare system [18], the government of Ghana stands to benefit greatly from knowing the actual gaps in its health delivery system in order to adopt data-driven policies to address them. Accordingly, the research examines the strengths and drawbacks of the coordination activities of RCCE in a selected FBHO during the outbreak of MVD in Ghana.

## Materials and method

The phenomenological research design was used to examine FBHO’s RCCE response to MVD outbreak in Ghana [3, 19]. We limited the scope of the research to the facility level to enable the study to be conducted within a predefined time and budget. Ethical approval was secured from the Ghana Health Service Institutional Review Board under review number GHS-ERC 007/02/23. Both written and informed consent was obtained from all participants.

### Participant selection and data collection

The research was conducted in the Christian Health Association of Ghana (CHAG). CHAG is the largest local implementation partner of the Ghana Health Service. The health facilities of CHAG are owned and managed by 34 Church denominations. CHAG is also the second largest health service provider in Ghana with 34,589 employees and 374 facilities delivering primary, secondary, and tertiary healthcare services. It currently serves at least 11,308,640 people in Ghana [18]. The target facility is a Primary Hospital and the only referral facility in the district. The hospital’s total OPD attendance in 2022 was 26,972, bed capacity 41, available bed days 14,965, and annual inpatient days 7,775. The target population consists of both clinical and nonclinical healthcare professionals who were directly involved in the management of MVD in Ghana. The purposive sampling technique was employed to select participants whose descriptions and experiences are relevant to the research question. We obtained authorisation from CHAG to enter the facility, and the participants contacted through phone calls to participate in the study after their consents were taken. We interviewed 15 participants using a semi-structured interview guide with open-ended questions. The number of interviews conducted was determined by data saturation [19]. The cadres include the District Deputy Chief Disease Control Officer, District SNO Public Health, Hospital Administrator, Senior Health Service Administrator, Senior Medical officer, Medical Director, Medical Doctor, Nurse Manager, Nursing Officers, Enrolled Nurse, Biomedical Scientist and Human Resource Manager. The guide had three sections, namely participants’ Demographic characteristics of participants, emergency preparedness and response and collaborations with other stakeholders. The interviews were conducted virtually on Zoom in the English language between 20^th^ April 2023 to 1^st^ August 2023 and recorded. The video files were transcribed, cleaned, coded and analysed with NVivo (version 14) [19]. To ensure that participants’ responses were not influenced by the work environment and/or colleagues, each participant was required to isolate himself/herself during the interviews. Where isolation was not possible, participants were interviewed after working hours. The participants provided written informed consent to take part in the research.

### Data analysis

Braun and Clarke’s [20] thematic analysis was employed to thematize and analyse data. The process involved coding and data organization at three levels: Similar responses and implied meanings were organized in two thematic areas. We then summarised similar ideas in each theme into subthemes. From this, specific information was deduced from the data to assess the institution’s RCCE capacity during the outbreak.

### Ethical considerations

Participants provided informed consent. To ensure confidentiality, identifiers such as facility names and residences were either substituted with pseudonyms or omitted in the publication.

## Findings

Table 1 shows the demographic characteristics of participants. These include a District Deputy Chief Disease Control Officer, a District SNO Public Health, a Hospital Administrator, a Senior Health Service Administrator, a Senior Medical officer, a Medical Director, a Medical Doctor, a nurse manager, 4 nursing officers, an enrolled nurse, one biomedical scientist and a human resource manager. There were more females (n = 9) in the study than males (n = 6). Also, the majority were aged between 30 and 39 years (n = 9), followed by those between 40 and 49 years (n = 4). Eleven participants were married at the time of the research and 12 were parents.

**Table 1:** Details the demographic characteristics of research participants.

NAME	DESIGNATION	AGE (YEARS)	GENDER	MARITAL STATUS	PARENTAL STATUS	YEARS OF EMPLOYMENT
DDCO	District Deputy Chief Disease Control Officer	44	M	Married	Yes	2
DPHN	District SNO, Public Health	36	F	Married	Yes	5
HA	Hospital Administrator	43	M	Married	Yes	14
SHSA	Senior Health Service Administrator	34	F	Married	Yes	5
SMO	Senior Medical Officer	37	F	Married	Yes	4
MDir	Medical Director	42	M	Married	Yes	16
MD	Medical Doctor	46	M	Married	Yes	10
NM	Nurse Manager	37	F	Married	No	4
NO.1	Nurses Officer	29	F	Single	Yes	7
NO.2		36	F	Single	Yes	10
NO.3		34	F	Married	Yes	8
BO 4		39	F	Married	Yes	8
EN	Enrolled Nurse	26	F	Single	No	3
BS	Biomedical Scientist	31	M	Single	No	4
HRM	Human Resource	38	M	Married	Yes	5

Source: Field data, 2023

Table 2 highlights the main theme and subthemes from the thematic analysis conducted. It identified RCCE as the focus of the study. Community engagement, capacity building and managing disinformation; Risk communication (Early warning systems); and Stakeholder collaboration for RCCE are the subthemes.

**Table 2:** Emerging themes.

RESEARCH OBJECTIVE	THEMES	SUB-THEMES
To examine the emergency preparedness and response capacity for RCCE in CHAG facilities	RCCE	• Community engagement and Capacity building and Managing disinformation. • Risk communication (Early warning systems) • Stakeholder collaboration for RCCE

Source: Field data, 2023

### Community engagement and infodemic management

The participants described three levels of communication. This includes data sharing at the health human resource level, at the community and patient level, and the institutional level. Many of them were satisfied with the processes adopted to communicate the outbreak with the staff of the hospital and partner agencies. However, the engagement with patients on admission was not considered the best. We also observed that while majority were aware of the facility’s engagement with the community, the frontline workers were not actively involved in the process. As a result, disinformation was still prevalent.

On community engagement, capacity building and control of disinformation regarding MVD, some of the participants had this to say:

*An emergency meeting was conducted by management. We also wrote letters to the district, region and other necessary places to request for staff to come and help (HR).*

*We first meet the HODs and had a meeting about the conditions. And then we printed out information about the outbreak and pasted it on our walls. We started educating our patients through that came to the facility encouraging them to protect themselves against the condition… Also, when a patient is brought in by a taxi driver, we go to the driver and educate the driver about infection prevention, so we take our chlorine solution to the car and disinfect the car (NM)*.

*We went to information centers, CHPS Compounds and the health centers around so that they will also change their referring strategies (MD)*.

*My director and the disease control officer were regularly going to the houses of patients*, *or calling them on the phone to ask how they were doing*. *If they are experiencing any sign and symptoms*, *we bring them in with vehicles to avoid stigmatizing them*. *The GHS staff were also going there and visiting relatives…We involved hunters*, *farmers those who deal in bush meat and all that*, *in risk communication*, *so we went to all the sub-district and brought them to one particular point and we explained the Marburg disease and how they are supposed to handle the meat and what they are supposed to do in case a hunter goes into the farm and sees an animal is dead… The facility is our district hospital*, *so during the district directorate’s performance review we invite them (DPHN)*.

### Coordinated and informal approaches to risk communication

Participants highlighted the use of both formal and informal communication channels to manage risk and engage the community effectively. Formal strategies included training frontline workers on case definitions, symptoms, and preventive measures, disseminating information through facility WhatsApp groups and community information centres, and conducting emergency meetings for unit representatives. Informal methods involved personal interactions with district health officials to facilitate rapid response and information flow. The lack of counselling services for mentally distressed staff was noted, with recommendations to seek support from colleagues and supervisors. Infection prevention measures were emphasized through posters and available PPE. Participants described the processes adopted for risk communication, and other early warning systems for distress management as follows:

*There isn’t any counseling for staff with mental distresses, but I think we were told that we should keep talking to relative and other colleague staff any time we feel traumatized. Staff were also advised to talk to my boss, myself, or the nurse manager. Prior to the quarantine individuals were called for routine checkups… we have facility WhatsApp pages as well. Information were sent to the pages. With regards to the community, the facility used information centers to announce the situation so that they may also be aware (MD).*

*All the frontline workers at all levels of healthcare delivery in the districts were trained on case definition, signs and symptoms identification, and what they are supposed to do. We also stressed on wearing mask, washing hands frequently, the use of sanitizers, among others. But I cannot say much about awareness creation among in-patients. Maybe the facility doesn’t want to create panic among clients. But there were posters on the disease, sanitizers, chlorine solutions, nose mask, gloves and so on for infection prevention and control activities. (DPHN)*.

*On a personal note, I often approach to district health director, disease control officer, district nutrition officer and district public health nurse informally, particularly during emergencies …We talk informally to arrange meetings and get needed information even before the official notices arrive. Because we realized that sometimes you will receive a letter today requiring action today. As such, when there is an emergency we first resort to informal methods (MDir)*.

*We give positive feedback and inputs to the district directorate on how to go about things because we are the district facility. We provide them with the necessary data (HA)*.

*Emergency meeting was called for representative of each unit in management. This was done at the management level to avoid crowding. We then had to disseminate the messages down to the rest of the staff. So there wasn’t a gathering of all staff. After this, the nurse manager took advantage of it to train nurses, inviting the disease control officer and medical director to participate in the training. They made sure the necessary training was out there for everybody to benefit but we tried to avoid overcrowding (HR)*.

*If we have to take samples to the lab, we inform the district director, the disease control officer, and other relevant officers (SMO)*.

*We educated the staff at the lab after Marburg was detected*, *because the lab was at the time testing samples for viral hemorrhage fever*. *So once Marburg was detected*, *I had to inform the lab to be careful (BS)*.

### Stakeholder collaboration for risk communication

Participants described a collaborative yet sometimes challenging relationship between their facility and various health and civic organizations. They highlighted regular support and meetings with the District Health Directorate, the Ghana Health Service, and other health facilities, fostering cooperation during public health emergencies like COVID-19 and Marburg outbreaks. Coordination included logistical support and policy development. However, conflicts arose over duplicated instructions and perceived inequities in resource distribution. Community engagement involved training nurses and volunteers, and working with opinion leaders, while logistical support often came through denominational offices. Despite challenges, efforts to maintain positive inter-organizational relationships were emphasized. When asked to describe the facility’s relationship with other agencies during the outbreak, some of them had this to say:

*The facility is attached to the District Directorate. District Director has been helping us in all activities, he comes regularly for meetings and discussions, and if there are challenges, he helps us and I go there for meetings. I present my public health issues to him and follow up on him for vaccination and all that. It was the disease control team and the biomedical scientists that we regularly worked with in sending samples. It has been like that throughout the COVID time. We also have working relationship with other health facilities in the district. We call on them when we have one or two challenges to deal with…Also, CHAG has a positive working relationship with GHS, because in almost all GHS programmes, we receive invitation to attend. Somewhere last year GHS, NCHS, CHAG, the MoH, and the director of nursing at MoH had a meeting and we represented and helped in drawing some policies for nurses. So I believe there’s a cordial relationship. With the Marburg, the Regional Health Director actually came to the facility to meet the management team. The National Public Health department also came and they made us prepare a logistic request through the district so they brought us some items like PPEs (HA).*

*We had NCCE sending some people over. We also had reps from Ghana Health Service surveillance unit in Accra. The National Commission for Civil Education was contacted by the district health directorate to create awareness in the district. I would add that our relationship with the District Health Directorate is not all rosy. Sometimes, there has been conflicts because they would instruct your staff on our blind side, often duplicating operational processes. Again, when it comes to distribution of assets or medical equipment or consumables, we feel that for the cadre of staff and the number of patients we see, they don’t share the items based on data. So it’s not all rosy (MDir)*.

*The CHAG denominational secretary has always been around. Anytime the District Director was coming, they came with him and resources allocated to us comes through the denominational office (HR)*.

*Often*, *during public health emergencies*, *other facilities in the district are invited to work together*. *We have trained the nurses and volunteers in the community to fish out those who are showing the symptoms… When it comes to the community*, *we normally liaise with opinion leaders like churches*. *The nurses go there for case search and visit both the passive and active cases*, *and when necessary*, *call us (DDCO)*.

## Discussion

The study adapted the COVID-19 Strategic Preparedness and Response Plan for the WHO African Region (2021) to evaluate the Risk Communication, Community Engagement, and Infodemic Management (RCCE) efforts of a CHAG-member facility in addressing disinformation and infodemics during Marburg disease outbreaks in Ghana [5].

RCCE uses evidence-based approaches to limit the impact of disease outbreaks on communities and individuals. The tool builds the willingness of communities and individuals through community engagement, risk communication and other chains of communication strategies to reduce infections. It is a bottom-up approach for reinforcing the capacity of communities, clinical and non-clinical professionals, non-governmental organizations, and the media to resist infodemics and have a management response system to counter disinformation [3, 5].

The findings of the study highlight several aspects of the capacity of faith-based organizations (FBOs) for Risk Communication, Community Engagement, and Infodemic Management (RCCE) during outbreaks. The engagement of multiple stakeholders emerged as a significant aspect of the facilities’ strategies to achieve this goal. Participants highlighted engagement with various stakeholders, including the National Commission for Civic Education (NCCE), Ghana Health Service (GHS), Ministry of Health (MoH), Executive Office of the Christian Health Association of Ghana (CHAG), World Health Organization (WHO), community champions, District Health Directorate, and other health facilities [21]. It was also observed that the denominational secretary’s consistent presence and the allocation of resources through the denominational office highlighted the integral role of CHAG in facilitating support during public health emergencies. However, although there is evidence of multi-level communication strategies, gaps in effective engagement at crucial touchpoints persist, contributing to the likelihood of disinformation.

In terms of risk communication, the participants reported a three-tiered approach involving communication at the health human resource level, patient level, and institutional level [3, 5]. While satisfaction was expressed regarding communication with hospital staff and partner agencies, a noticeable shortfall is evident in patient engagement upon admission. This gap might stem from a lack of active involvement of frontline workers in the community engagement process, allowing disinformation to thrive [2]. The consequence is the persistence of misinformation within the community.

Further, community engagement emerged as a crucial component of RCCE, with participants describing various strategies to build capacity and control disinformation. Emergency meetings at the management level were conducted, and letters were sent to relevant authorities to request additional staff. Additionally, information about the outbreak was disseminated within the facility through printed materials, emphasizing patient education and infection prevention [3]. Collaborative efforts were made to extend outreach beyond the facility, targeting information centres, CHPS Compounds, and health centres. The involvement of diverse community members, including taxi drivers, hunters, farmers, and those dealing with bush meat, demonstrated a comprehensive approach to risk communication. Despite these efforts, gaps in community engagement were identified. The study revealed that while the facility’s directorate engaged with the community during performance reviews, frontline workers were not actively participating in the process. This lack of involvement may contribute to gaps in information dissemination and hinder the effectiveness of risk communication efforts. Furthermore, the study indicated a need for continuous counselling and distress management for staff, especially in the absence of formal mechanisms for mental health support [2, 3].

Additionally, the mechanisms employed for risk communication and distress management showed variation across different levels of healthcare delivery. While efforts were made to disseminate information through various channels including facility WhatsApp pages and information centres, the absence of formal counselling services for staff facing mental distress is a critical gap. The reliance on informal support networks, though a commendable interim measure, may not sufficiently address the psychological impact on staff during crises [3]. Additionally, the varied emphasis on training frontline workers across healthcare delivery levels is observed. Frontline workers received training on case definition, identification of signs and symptoms, and preventive measures, awareness creation among in-patients in a limited scope to avoid panic. While the training was widespread among various cadre of workers, there appears to be a cautious approach to creating awareness among in-patients, potentially impacting the depth of understanding within this crucial demographic [2, 5].

Informal communication channels played a significant role in RCCE processes. Participants emphasized the importance of informal interactions with district health directors, disease control officers, and other relevant authorities during emergencies. The proactive involvement of FBOs in providing feedback, sharing data, and coordinating with district directorates showcases a commitment to a cohesive response [3, 5]. This informal approach enabled timely access to information and coordination of actions. However, the study identified a potential risk in relying solely on informal methods, as formal notices and actions might be delayed.

In conclusion, the research revealed commendable achievements as well as intricate challenges with RCCE during the Marburg disease outbreak. The Risk Communication, Community Engagement, and Infodemic Management (RCCE) mechanisms adopted showcase effective strategies along with gaps in frontline worker involvement, formal counselling for staff, and coordination between communication channels. The imperative to upskill frontline workers, formalize mental health support, refine patient engagement, and establish structured communication channels emerges as a critical focus for fortifying RCCE capacities.

## Limitations

The study has inherent limitations. Interviews were conducted one year after the MVD outbreak in Ghana, potentially leading to recall bias and forgetfulness. To address this, a concise background summary preceded substantive questions for each interviewee. Additionally, the scope of the research was confined to perspectives of healthcare workers on risk communication and community engagement efforts during the outbreak, without direct input from community members and clients. Future research should therefore focus on understanding the experiences of communities and patients, seeking their opinions and recommendations.

## Conclusion

The study reveals both strengths and weaknesses in the capacity of faith-based organizations for RCCE during disease outbreaks [3]. Effective risk communication and community engagement strategies were employed, but gaps in the involvement of frontline workers, formal counselling for staff, and coordination between formal and informal communication channels were evident. Other gaps include insufficient patient engagement, limited involvement of frontline workers in community engagement, absence of formal mental health support for staff, and varying degrees of awareness creation among in-patients. The future trajectory necessitates a comprehensive approach that involves upskilling frontline workers, formalizing mental health support mechanisms, refining patient engagement strategies, and establishing structured communication channels with health authorities. FBOs need to integrate these improvements into their protocols to bridge existing gaps effectively and fortify their capacity for robust RCCE, thereby combating the rampant infodemic in public health emergencies.

## References

[1] Akande OW, Disu Y, Kaduru C, Anueyiagu C, Oguanuo E, Ojumu T, et al. Risk communication during health emergencies in Nigeria: What are its challenges? J Public Health Afr [Internet]. 2023 Jan 1 [cited 2024 Jan 14];14(1). Available from: /pmc/articles/PMC9926553/ doi: 10.4081/jphia.2023.1943 36798846 PMC9926553

[2] Aboagye AK, Dai B, Bakpa EK. The effect of risk communication on the nurses’ task and contextual performance in disease outbreak control in Ghana: Application of the cause model. International Journal of Health Planning & Management [Internet]. 2020;35(4):922–38. Available from: https://www.ezproxy.is.ed.ac.uk/login?url= http://ovidsp.ovid.com/ovidweb.cgi?T=JS&CSC=Y&NEWS=N&PAGE=fulltext&D=med18&AN=32323897 doi: 10.1002/hpm.2978 32323897

[3] A risk communication, community engagement and infodemic management toolkit for mpox elimination: 17 May 2023 update [Internet]. [cited 2024 Jul 12]. Available from: https://iris.who.int/handle/10665/367853

[4] Risk Communication and Community Engagement for Ebola Virus Disease Preparedness and Response—Lessons Learnt and Recommendations from Burundi, Rwanda, South Sudan, Tanzania and Uganda—Democratic Republic of the Congo | ReliefWeb [Internet]. [cited 2024 Jan 14]. Available from: https://reliefweb.int/report/democratic-republic-congo/risk-communication-and-community-engagement-ebola-virus-disease

[5] Strategic Response to COVID-19 in the WHO African Region (Update—April 2021) | WHO | Regional Office for Africa [Internet]. [cited 2024 Jul 12]. Available from: https://www.afro.who.int/publications/strategic-response-covid-19-who-african-region-update-april-2021

[6] Wang Y, Hao H, Platt LS. Examining risk and crisis communications of government agencies and stakeholders during early-stages of COVID-19 on Twitter. Comput Human Behav. 2021 Jan 1;114:106568. doi: 10.1016/j.chb.2020.106568 32982038 PMC7508681

[7] Best practices for communicating with the public during an outbreak Outbreak Communication [Internet]. Available from: http://www.who.int/csr

[8] Frimpong SO, Paintsil E. Community engagement in Ebola outbreaks in sub-Saharan Africa and implications for COVID-19 control: A scoping review. International Journal of Infectious Diseases. 2023 Jan 1;126:182–92. doi: 10.1016/j.ijid.2022.11.032 36462575

[9] Gilmore B, Ndejjo R, Tchetchia A, De Claro V, Mago E, Diallo AA, et al. Community engagement for COVID-19 prevention and control: a rapid evidence synthesis. BMJ Glob Health [Internet]. 2020 Oct 13 [cited 2024 Jan 14];5(10):3188. Available from: /pmc/articles/PMC7554411/ doi: 10.1136/bmjgh-2020-003188 33051285 PMC7554411

[10] Adebisi YA, Rabe A, Lucero-Prisno DE. Risk communication and community engagement strategies for COVID-19 in 13 African countries. Health Promot Perspect [Internet]. 2021 [cited 2024 Jan 14];11(2):137. Available from: /pmc/articles/PMC8233683/ doi: 10.34172/hpp.2021.18 34195037 PMC8233683

[11] Boulenger D, Barten F, Criel B. CONTRACTING BETWEEN FAITH-BASED HEALTH CARE ORGANIZATIONS AND THE PUBLIC SECTOR IN AFRICA. Review of Faith and International Affairs [Internet]. 2014 Jan [cited 2024 Jan 5];12(1):21–9. Available from: https://www.tandfonline.com/doi/abs/10.1080/15570274.2013.876730

[12] Peprah P, Budu HI, Agyemang-Duah W, Abalo EM, Gyimah AA. Why does inaccessibility widely exist in healthcare in Ghana? Understanding the reasons from past to present. Journal of Public Health (Germany) [Internet]. 2020 Feb 1 [cited 2024 Jan 5];28(1):1–10. Available from: https://link.springer.com/article/10.1007/s10389-019-01019-x

[13] COVID-19 Strategic Preparedness and Response Plan for the WHO African Region, 1 February 2022–31 March 2023 (Updated on 31 July 2022) | WHO | Regional Office for Africa [Internet]. [cited 2024 Jan 1]. Available from: https://www.afro.who.int/publications/covid-19-strategic-preparedness-and-response-plan-who-african-region-1-february-2022

[14] Ngoy N, Oyugi B, Ouma PO, Conteh IN, Woldetsadik SF, Nanyunja M, et al. Coordination mechanisms for COVID-19 in the WHO Regional office for Africa. BMC Health Serv Res [Internet]. 2022 Dec 1 [cited 2024 Jan 5];22(1):1–17. Available from: https://link.springer.com/articles/10.1186/s12913-022-08035-w 35643550 10.1186/s12913-022-08035-w PMC9142827

[15] Grieve A, Olivier J. Towards universal health coverage: A mixed-method study mapping the development of the faith-based non-profit sector in the Ghanaian health system. Int J Equity Health [Internet]. 2018 Oct 5 [cited 2024 Jan 5];17(1):1–20. Available from: https://equityhealthj.biomedcentral.com/articles/10.1186/s12939-018-0810-4 30286758 10.1186/s12939-018-0810-4 PMC6172851

[16] Bonney JK, Adu B, Sanders T, Pratt D, Adams P, Asante IA, et al. Marburg Virus Disease in Ghana. New England Journal of Medicine [Internet]. 2023 Jun 22 [cited 2024 Jan 1];388(25):2393–4. Available from: http://www.nejm.org/doi/10.1056/NEJMc2300867 37342928 10.1056/NEJMc2300867

[17] Denkyira SA, Adesola RO, Idris I, Yelarge K, Tieku Asaaseasa K, Danquah CA, et al. Marburg virus in Ghana: A public health threat to Ghanaians and to Africans. Public Health Challenges. 2022 Dec;1(4). 10.1002/puh2.32 PMC12039705 40496679

[18] CHAG Home–Christian Heallth Association of Ghana [Internet]. [cited 2024 Jan 2]. Available from: https://chag.org.gh/

[19] Creswell J. W., & Poth C. N. (2016). Qualitative inquiry and research design: Choosing among five approaches. Sage publications.—Google Search [Internet]. [cited 2024 Jan 1]. Available from: https://www.google.com/search?q=Creswell%2C+J.+W.%2C+%26+Poth%2C+C.+N.+(2016).+Qualitative+inquiry+and+research+design%3A+Choosing+among+five+approaches.+Sage+publications.&rlz=1C1GCEA_enGH1002GH1002&oq=Creswell%2C+J.+W.%2C+%26+Poth%2C+C.+N.+(2016).+Qualitative+inquiry+and+research+design%3A+Choosing+among+five+approaches.+Sage+publications.&gs_lcrp=EgZjaHJvbWUyBggAEEUYOdIBCDE4NDZqMGo3qAIAsAIA&sourceid=chrome&ie=UTF-8

[20] Braun V, Clarke V. Using thematic analysis in psychology. Qual Res Psychol. 2006;3(2):77–101.

[21] Vanderslott S, Van Ryneveld M, Marchant M, Lees S, Nolna SK, Marsh V. How can community engagement in health research be strengthened for infectious disease outbreaks in Sub-Saharan Africa? A scoping review of the literature. BMC Public Health. 2021 Dec 1;21(1). doi: 10.1186/s12889-021-10348-0 33794820 PMC8012744

